# Sylvia J. T. Jansen, Henny C. C. H. Coolen and Roland W. Goetgeluk (eds): The measurement and analysis of housing preference and choice: Springer, 2011, 272 pp, €99.95, ISBN 978-90-481-8893-2

**DOI:** 10.1007/s10901-012-9287-4

**Published:** 2012-04-01

**Authors:** H. J. P. Timmermans

**Affiliations:** Urban Management and Design Systems, Technische Universiteit Eindhoven, Eindhoven, The Netherlands

This book brings together 11 contributions—all by scholars based at the Delft University of Technology, The Netherlands but from different faculties—on alternative measurement approaches and models of housing preference and choice. The volume is, however, more than an arbitrary collection of individual chapters in that every chapter attempts to address a set of common issues. In addition to an explanation of the methods and their limitations, attention is paid to applications, which makes the book valuable for educational purposes.

The approaches range from widely applied conjoint measurement methods to methods such as decision plan nets, which despite their potential have never found much following in housing research. Yet others, such as residential images, seem parochial. More specifically, after introductory chapters by the editors of the book and by Boumeester on traditional housing demand research, Goetgeluk discusses the principles of decision plan nets, and Coolen expands on the meaning structure method, which bears much resemblance to familiar laddering techniques. Jansen then continues with an introduction to multi-attribute utility theory, followed by Molin’s chapter on conjoint analysis. Singelenberg et al. then continue with a discussion of the residential images method, followed by Jansen on the lifestyle method. This chapter is slightly off topic, in the sense that lifestyle is not really a method and does not come with a succinct methodology. However, the chapter does give a detailed overview of the use of the concept of lifestyle and its limitations in housing research. After these ‘stated’ methods, Koopman discusses the neo-classical view of the housing market and two revealed preference methods, followed by de Groot on longitudinal analysis. Finally, the editors draw some overall conclusions.

In general, the level of the discussion is kept to the basics of the approach. The latest developments are not discussed at all or only receive scant attention, as in Molin’s chapter on stated preference and choice analysis. This makes the book less appropriate for understanding the most recent advances in the measurement and analysis of housing preferences and choice. Another missed opportunity is that the editors do not emphasize the similarities/overlap and potential combinations of the various measurement approaches. For example, conjoint analysis can be viewed as an elaboration/application of multi-attribute utility theory, whereas the chapters give the impression that these are unrelated methods. Similarly, under the right set of conditions, the residential images approach can be and has been linked to conjoint analysis.

Despite these limitations, the volume is unique in its kind. It brings together explanations and case studies of a wide variety of methods that have been used in both academic and applied research to measure and analyze housing preferences and choices. The comprehensive introductory discussion of the methods and the detailed applications make the book valuable reading for classes offering an introduction to housing research methods in disciplines such as geography, planning and real estate for academics and other professionals. Because housing research tends to be fragmented in terms of different networks using different methods, the book may also serve as an eye-opener for those researchers and other professionals who have knowledge and experience drawn only from a particular approach.

